# 12Fr-Pigtail Versus 14Fr-Balloon Percutaneous Radiologic Gastrostomy (PRG), Retrospective Evaluation of Outcomes and Complications; A Maastricht University Medical Centre Study

**DOI:** 10.1007/s00270-023-03527-6

**Published:** 2023-08-17

**Authors:** Glenn Dams, Robrecht R. M. M. Knapen, Remon Korenblik, Ronald M. van Dam, Michiel W. de Haan, Christiaan van der Leij

**Affiliations:** 1 Department of Radiology and Nuclear Medicine, Zuyderland MC, Sittard-Geleen, Netherlands; 2 Department of Radiology and Nuclear Medicine, Maastricht University Medical Center+, Maastricht, Netherlands; 3 CARIM-School for Cardiovascular Diseases, Maastricht University, Maastricht, Netherlands; 4 Department of Surgery, Zuyderland MC, Sittard-Geleen, Netherlands; 5 GROW-School for Oncology and Developmental Biology, Maastricht University, Maastricht, Netherlands; 6 Department of Surgery, Maastricht University Medical Center+, Maastricht, Netherlands; 7 Department of General, Visceral and Transplant Surgery, University Hospital Aachen, Aachen, Germany

**Keywords:** Percutaneous radiologic gastrostomy (PRG), Percutaneous endoscopic gastrostomy (PEG), 14Fr-balloon tubes, 12Fr-pigtail tubes

## Abstract

**Purpose:**

To retrospectively compare tube and placement related results of a 12Fr-pigtail and a 14Fr-balloon gastrostomy tube.

**Materials and Methods:**

All consecutive patients who underwent percutaneous radiologic gastrostomy (PRG) between January 2016 and June 2020 were enrolled in this retrospective single-center analysis. Follow-up for all patients was 180 days. Mortality after 30 days, technical success, days to first complication within 180 days, reason of unexpected visit (tube, anchor or pain related), and tube specific complications (obstruction, pain, luxation, leakage) were taken as outcome measures. Data were obtained from both PACS software and electronic health records.

**Results:**

A total of 247 patients were enrolled (12Fr-pigtail: *n* = 139 patients and 14Fr-balloon: *n* = 108 patients). 30-day mortality was very low in both groups and never procedure related. Technical success was 99% in both groups. The average number of complications within 180 days after initial PRG placement was significantly higher in the 12Fr-pigtail group (12Fr-pigtail: 0.93 vs. 14Fr-balloon: 0.64, *p* = 0.028). Time to first complication within 180 days was significantly longer in the 14Fr-balloon group (12Fr-pigtail: 29 days vs. 14Fr-balloon: 53 days, *p* = 0.005). In the 14Fr-balloon group, the rate of tube-related complications (luxation and obstruction) was significantly lower compared to 12Fr-pigtail (29% vs. 45%, *p* = 0.011).

**Conclusion:**

14Fr-balloon gastrostomy tubes have significantly lower (tube-related) complications rates and longer time to first complication compared to 12Fr-pigtail tubes. No procedure-related mortality was observed in either group. Technical success was very high in both groups.

*Level of Evidence* Level 3, non-controlled retrospective cohort study.

## Introduction

Patients with a (neurological) swallowing disorder or a (malignant) stenosis in the pharynx or esophagus may develop inadequate oral nutritional uptake leading to secondary malnutrition. For these patients, enteral or parental feeding is often indicated to optimize the patient’s condition [1, 2].

Percutaneous gastrostomy is the placement of a feeding tube directly through the abdominal wall into the stomach, creating an artificial enterocutaneous fistula [2]. Percutaneous gastrostomy tubes are preferred to nasogastric tubes when longer-term enteral nutrition is necessitated, as they are more comfortable and have lower tube-related complications rates [2–4].

The gastrostomy tubes can be placed either surgically (by laparotomy), endoscopically [percutaneous endoscopic gastrostomy (PEG)], or percutaneously [percutaneous radiologic gastrostomy (PRG)]. Almost every patient with an indication for gastrostomy placement is eligible for PRG or PEG placement. However, sometimes PRG is preferred over endoscopic placement, for example in patients with head and neck cancer, cerebrovascular events, or neurological disorders [5].

Contraindications for PRG placement are percutaneously inaccessible stomach (due to hepatosplenomegaly or interposed intestines), presence of major esophageal varices, or coagulopathy. Innovations, such as changes in placement techniques and materials used, have led to fewer contraindications [6].

Recently, a single center retrospective study concluded that PEG and PRG showed equal results in respect to procedure-related and 30-day mortality rates [7], but lower tube-related complications in the PEG group compared to PRG. To explain this difference, the authors postulated that the higher tube-related complications were probably due to the smaller diameter of the PRG feeding tube and/or differences in fixation methods. As these pigtail-retaining catheters solely depend on a wire within the tube that locks the tube into a loop (in the stomach), the dissolvement of the wire in the acidic gastric environment could lead to relaxation of the loop and subsequently dislodgement of the tube [8].

Shortly after publications, the 12Fr-pigtail tubes were abandoned and replaced in our practice by wider diameter (14Fr) balloon tubes. The balloon retention mechanism, incorporated into the tube, inflates inside the stomach. This fixation technique is supposed to be more reliable [5].

Considering these differences, the aim of this study was to compare the 14Fr-balloon catheter and 12Fr-pigtail catheter systems regarding successful rate, mortality and complications.

## Materials and Methods

### Research Design

In this retrospective cohort study, all consecutive patients, who underwent PRG placement at Maastricht University Medical Centre between January 1 2016 and June 1 2020, were included. Data from electronic health records and PACS system of all patients were retrospectively analyzed and collected into an online database [Castor EDC (2019)]. Ethical approval was obtained (METC 2020-2246), and data were collected in a pseudonymized fashion.

Patients who received initial PRG placement using the 12Fr (pigtail type) Wills-Oglesby Percutaneous Gastrostomy Set (Cook Medical, Bloomington, Indiana, United States) or using the 14Fr (balloon type) Entuit Gastrostomy (Cook Medical)/Flocare Gastrostomy tubes (Nutricia, Amsterdam, North Holland, The Netherlands) were included. Except for patients who died within 30 days, patients with less than 6 months follow-up were excluded.

Medical records were used to collect the baseline characteristics. Radiological reports and letters within patient files were used to check for complications related to the PRG placement. Visits were categorized as expected or unexpected visits, based on reason for visit. Reasons for unexpected visit were categorized into tube-related, anchor-related or other. Outcome measures included technical success rate, mortality at 30 days, days between the initial tube placement and the first unexpected visit, the total number of unexpected visits per patient within 6 months after placement, and reasons for the unexpected visits (as described above). Tube related complications included tube leakage (defined as fluid along the drain) and tube repositioning, defined as replacement of the PRG based on clinical request (e.g., for malposition).

### Procedure

According to the CIRSE Safety checklist [9], (contra-) indications were checked preprocedural. Both the 12Fr-pigtail and the 14Fr-balloon catheter were placed using the standard placement methods with nasogastric tube inserted before the procedure (on the ward). Direct preprocedural time-out procedure was performed. The location of the stomach and possible interposing abdominal organs (liver, intestines) were identified using ultrasound and fluoroscopy. After intravenous injection of 20 mg Buscopan, the stomach was inflated through the nasogastric tube. Local anesthesia with 20–30 ml lidocaine 1% at the puncture site was injected. No prophylactic antibiotics or sedatives were administered pre- or periprocedural. Fluoroscopic and/or ultrasound guidance was used to puncture the stomach and to place the three anchors (Entuit™ Secure, Cook Medical). The anchors were placed in a triangular orientation, after checking the needle position using contrast material. Between the three anchors, the stomach was punctured, and a stiff guidewire inserted. The tract was dilated and a 12Fr- or 14Fr-tube was placed over the guidewire (the 14Fr-tube through an 18Fr peel-away sheath). The next day, the tube was flushed for 3 to 6 hours using saline fluids to check for complications. If no complications were observed, the tube could be used for enteral feeding and the patient was discharged. Routinely, after 10 to 14 days anchors were removed.

### Data Analysis

Baseline characteristics were described using standard statistics. Dichotomous or ordinal parameters were tested, using a chi-square test or Fisher’s exact test. A cox-regression/survival plot was conducted to check the outcomes of the days between initial placement and first unexpected visit between the groups and was presented with the hazard ratio with 95% confidence interval (CI). An alpha of 0.05 was respected. The statistical analyses were conducted with IBM SPSS Statistics 28 (IBM Corp. Version 28.0).

## Results

**Fig. 1:**
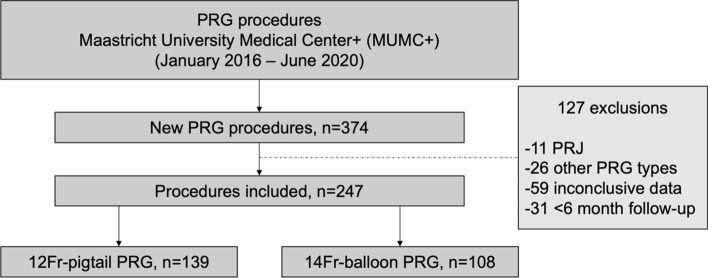
Flowchart of enrolled cases. *PRG* Percutaneous radiologic gastrostomy; *Fr* French; *PRJ* Percutaneous radiologic jejunostomy

A total of 374 procedures related to PRG-placements were performed between January 1 2016 and June 1 2020. After exclusion, 247 PRG placements were included: 12Fr-pigtail *n* = 139 and 14Fr-balloon *n* = 108. Reasons for exclusion are described in Fig. 1. Baseline characteristics are shown in Table 1. Apart from the Body-Mass-Index (BMI), which was significantly higher in the 14Fr-balloon catheter group, there were no significant differences between both groups.

**Table 1:** Baseline characteristics of patients who received either the 12Fr- or 14Fr-PRG at MUMC +  between January 2016 and June 2020

	12Fr-pigtail PRG (*n* = 139)	14Fr-balloon PRG (*n* = 108)	*P* value
Female—(%)	56 (40)	37 (34)	0.332
Age (average in years)—(SD)	63 (10)	64 (11)	0.588
BMI (average in kg/m^2^)—(SD)	22.7 (4.5)	24.9 (5.0)	**0.011**
Clinical indication—(%)			0.678
Head- or neck malignancy	101 (73)	72 (67)	
CVA and neurological disease	8 (5.8)	9 (8.3)	
Muscular disorders and ALS	12 (8.6)	13 (12)	
Other causes	18 (13)	14 (13)	
Comorbidities—(%)			
Heart and/or vascular disease	37 (27)	29 (27)	0.967
COPD	15 (11)	12 (11)	0.936
Hypertension	21 (15)	22 (20)	0.279
Diabetes	6 (4.3)	6 (5.6)	0.653
ASA classification—(%)			0.517
I	6 (4.3)	1 (0.9)	
II	55 (40)	43 (40)	
III	74 (53)	61 (57)	
IV	4 (2.9)	3 (2.8)	

Bold value indicate *p* < 0.05

*BMI* Body mass index, *CVA* Cerebral vascular accident, *ALS* Amyotrophic lateral sclerosis, *COPD* Chronic obstructive disease, *ASA* American society of anesthesiologist

### Technical Success Rate

Percentage successful placements were very high in both groups [12Fr-pigtail vs. 14Fr-balloon PRG, 99% and 99%, respectively (*p* = 1.00)]. In only one patient in both groups, the placement of a PRG was unsuccessful due to interposition of the intestines, both confirmed with fluoroscopy. Periprocedural complications occurred in two patients (one conservatively treated gastric perforation and one excessive pain) in the 12Fr-pigtail catheter group (< 2%) and in one patient (abdominal wall hematoma) in the 14Fr-balloon group (< 1%).

### 30-Day Mortality

The 30-day mortality was not significantly different between 12Fr-pigtail and 14Fr-balloon PRG, 2.2% (*n* = 3) and 0.9% (*n* = 1) (*p* = 0.634). All deaths were related to the underlying disease; none was procedure related.

### Days between Initial Placement and First Unexpected Visit

**Fig. 2:**
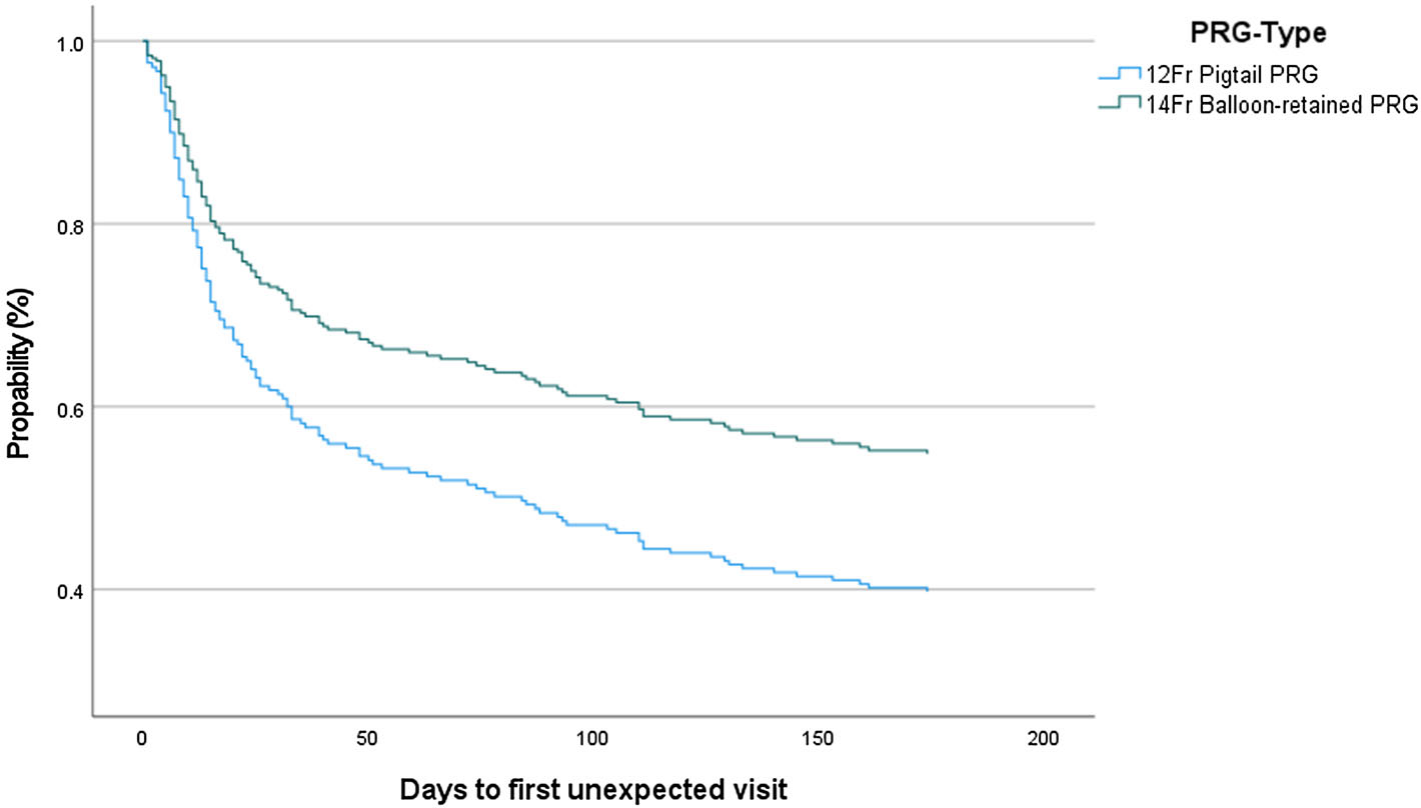
Cox regression survival curve. Number of days until first unexpected visit within first 180 days after initial PRG placement (*p* = 0.016). *PRG* Percutaneous radiologic gastrostomy

The first unexpected (PRG related) visit within 180 days after PRG placement occurred significantly less often in the 12Fr-pigtail group compared to the 14Fr-balloon group (*p* = 0.016, HR 0.65; 95% CI 0.46–0.93) (Fig. 2). When an unexpected visit occurred, the average number of days after PRG placement was significantly lower in the 12Fr-pigtail group compared to the 14Fr-balloon group (29 vs. 53 days, *p* = 0.005) (Table 2).

**Table 2:** Outcomes between patients who received the 12Fr- or 14Fr-PRG

All patients	12Fr-pigtail PRG (*n* = 139)	14Fr-balloon PRG (*n* = 108)	*P* value
Patients with at least one unexpected visit within 180 days—*n* (%)			
	82 (59)	50 (46)	**0.047**
Unexpected visits in 180 days—*n* (%)			0.162
0	57 (41)	58 (53.7)	
1	53 (38.1)	36 (33)	
2	17 (12.2)	10 (9.3)	
> 2	12 (8.6)	4 (3.7)	
Average unexpected visits within 180 days—mean (95% CI)			
	0.93 (0.74–1.12)	0.64 (0.48–0.80)	**0.028**
Days until first unexpected visit—mean (95% CI)			
	29 (22–36)	53 (38–68)	**0.005**
Reason unexpected visit—*n* (%)			
Tube complication	62/139 (45)	31/108 (29)	**0.011**
Anchor complication	11/139 (7.9)	8/108 (7.4)	0.882
Other*	9/139 (6.5)	11/108 (10)	0.289
Type of tube complication—*n*/total (%)			
Leakage	3/139 (2.2)	6/108 (5.6)	0.158
Obstruction	9/139 (6.5)	1/108 (0.9)	**0.046**
Luxation	45/139 (32)	20/108 (19)	**0.014**
Repositioning	3/139 (2.2)	4/108 (3.7)	0.702
Other^#^	2/139 (1.4)	0/108 (0)	0.506

Bold values indicate *p* < 0.05

^*^Reason for unexpected visit was in nine patients unknown, in seven patients position check, in two patients pain unrelated to anchors, in one patient blood around tube, and in one patient request for extending the tube

^#^In two patients the reason for tube replacement was unknown

### Total Number of Unexpected Visits

The number of visits per patient (expected and unexpected) is displayed in Table 2. Unexpected visits within the first 180 days were less frequent in the 14Fr-group (50 patients, 46%) compared to the 12Fr-group (82 patients, 59%), *p* = 0.047. The number of unexpected visits per patient between the groups was also significantly different (0.93 for 12Fr-pigtail and 0.64 for 14Fr-balloon, *p* = 0.028).

### Reasons for Unexpected Visits

All reasons for an unexpected visit are presented in Table 2. There were no significant differences between the number of anchor related or other related unexpected visits between the groups (*p* = 0.882 and *p* = 0.289, respectively). Tube-related complications were more often seen in the 12Fr-pigtail group (45% vs. 29%, *p* = 0.011). When looking at the tube related complications specifically, tube obstruction and luxation occurred more often in the 12Fr-pigtail group compared to the 14Fr-balloon catheter group [6.5% vs. 0.9% (*p* = 0.046) and 32% vs. 19% (*p* = 0.014)].

## Discussion

This single-center retrospective cohort study demonstrates that 14Fr-balloon gastrostomy tubes perform better in overall complication risk and time to first complication compared to 12Fr-pigtail tubes. Both groups showed equivalent results in terms of 30-day mortality and technical success rates.

Studies comparing different PRG types are scarce. One study compared three PRG techniques with 12-18Fr tubes over 25 years. The balloon type with gastropexy led to a decrease in complications [10]. In our study, PRG placement with anchor fixation was used. When looking at different diameters of the tubes, it has been previously described that 14Fr-tubes are more susceptible for tube complications compared to 20Fr-tubes [11]. In our study, the tube-related complications did significantly differ between the two groups, with higher rate of tube leakage and tube obstruction in the 12Fr-pigtail group. This confirms the initial thought that wider (14Fr) tubes and balloon retention mechanism perform better compared to 12Fr-pigtail type PRG tubes.

Recently, one study and one meta-analysis concluded that PEG and PRG showed equal results in respect to procedure-related and 30-day mortality rates but lower tube-related complications (obstruction and luxation) in PEG compared to PRG [7, 12]. Concluded was that tube-related complications were probably due to the smaller diameter of the PRG feeding tube and differences in fixation methods. In this study, 12Fr-pigtail tubes were used for PRG placement [7]. As our study showed a significantly lower obstruction and luxation rate in the 14Fr-balloon tube group compared to the 12Fr-pigtail tubes, this performance improvement is likely to level out the reported difference between PEG and PRG. Prospective comparative (multicenter randomized) trials should be performed to confirm this. A recent single-center randomized controlled trial already observed noninferiority of radiologically inserted gastrostomy compared to PEG [13].

Compared to the literature, we still observed a higher number of unexpected (PRG related) visits, and tube related complications in the 14Fr-balloon tube compared to the performance of PEG [7, 12]. This difference is explained by the fact that we registered complications over a period of 180 days after placement, as opposed to the 30 days used in most literature. Within the first 30 days, we registered 43 patients (17%) with an unexpected (PRG related) visit due to a tube related complication, showing equal or even lower numbers compared to Strijbos et al. [7].

When using larger PRG tubes, one could speculate that the use of larger sheaths for insertion of the tube (a 12 Fr-pigtail tube vs. a 18Fr peel-away sheath for the 14Fr-balloon tube) might increase the risk of periprocedural complications. The current study, however, still showed a very low (< 1%) periprocedural complication rate in the 14Fr-balloon group, comparable to the complication rate observed in the 12Fr group. This low rate, combined with the high technical success rate and the fact that the PRG tubes can be placed under local anesthesia, makes the radiological placement of a PRG a very safe, straightforward, and lean procedure, which would probably be reflected in the total costs. As a cost-effectiveness analysis was not a part of the current study, we were not able to conclude on this specific topic. Literature, however, already shows a favorable cost-profile of PRG compared to PEG [14].

At baseline, only BMI differed between the groups favoring the 12Fr-pigtail tube group. Nevertheless, due to the small differences with little clinical relevance, no significant influence was expected on the superiority of the 14Fr-balloon tube. Additionally, the recent literature showed no differences in complications rates and similar technical success between obese and non-obese patients [15].

Some limitations should be noted. First, the retrospective nature of the current study cannot preclude a selection bias. By including all consecutive patients, it was tried to minimize this effect. Second, this study is performed in a single center setting. This might influence the external validity of the results, as experience levels could be different in other hospitals. Third, the 14Fr-balloon tubes were mainly placed in recent years compared to the 12Fr-pigtail tubes, so an era bias cannot be ruled out as well. Procedural steps (except for the dilatation and placement of a peel-away sheath), however, were similar and not changed during the study period.

## Conclusion

14Fr-balloon gastrostomy tubes have significantly lower complications rates and longer time to first complication compared to 12Fr-pigtail tubes. No procedure-related mortality was observed in either group. The technical success was very high in both groups.
